# Effects of voltage-gated sodium channels on the median effective dose of ropivacaine in diabetic rats

**DOI:** 10.1038/s41598-026-49845-w

**Published:** 2026-05-09

**Authors:** Xueyin Song, Kang Lv, Peixia Yu, Xiang Liu, Yaozong Yu, Gaoya Cao, Yanlei Tai, Bo Zhao, Qiujun Wang

**Affiliations:** 1 Department of Anesthesiology, Hebei Medical University Third Hospital, Hebei Provincial Key Laboratory of Precision Anesthesia and Organ Protection, NO.139, Ziqiang Road, Shijia Zhuang, Hebei Province China; 2 Department of Neurosurgery, Hebei General Hospital, NO.348, Heping West Road, Shijia Zhuang, Hebei Province China; 3 Experimental Center for Teaching, Hebei Medical University, No.361, Zhongshan East Road, Shijia Zhuang, Hebei China

**Keywords:** Ropivacaine, Diabetes mellitus, Median effective dose, Voltage-gated sodium channels, Diabetic neuropathy, Diseases, Drug discovery, Medical research, Neurology, Neuroscience

## Abstract

Diabetes mellitus significantly impacts perioperative anesthetic management. Patients with diabetic neuropathy exhibit increased sensitivity to local anesthetics, yet the underlying mechanisms, particularly regarding the median effective dose (ED_50_) of ropivacaine and the role of voltage-gated sodium channels (Navs), remain incompletely elucidated. This study investigated the role of Navs in mediating the ED_50_ of ropivacaine in streptozotocin (STZ)-induced diabetic rats. Using the Dixon up-and-down method, the ED_50_ for sciatic nerve motor block was significantly lower in diabetic rats (0.100%) than in controls (0.142%), indicating heightened local anesthetic sensitivity. This pharmacodynamic shift was associated with underlying neuropathic changes, including reduced motor nerve conduction velocity (MNCV), decreased axon density, and downregulated expression of key Nav subtypes (Nav1.7, Nav1.8, Nav1.9) and the contactin-associated protein (CASPR) in the sciatic nerve and dorsal root ganglia (DRG). Crucially, administration of ropivacaine at the identified ED_50_ dose did not exacerbate neurophysiological dysfunction, histological damage, or alter Navs/CASPR expression in diabetic nerves compared with saline-treated diabetic group, demonstrating a favorable safety profile. These findings elucidate the mechanism underlying reduced local anesthetic requirements in diabetic neuropathy, which involves Navs downregulation and structural nerve alterations, and support the clinical use of lower, effective ropivacaine doses in diabetic patients to achieve successful analgesia without increasing neurotoxicity risk.

**Supplementary Information:**

The online version contains supplementary material available at 10.1038/s41598-026-49845-w.

## Introduction

Diabetes is the most common metabolic disease worldwide. Currently, there are approximately 463 million people with diabetes globally, with a prevalence rate of about 9.3%. In 2020, the elderly population aged 60 and above accounted for 18.7% of the total population (260.4 million), among which approximately 30% of the elderly were diabetic patients in China^1^. With changes in population structure, it is projected that the proportion of diabetic patients during the perioperative period will also continue to rise^2,3^. Because diabetes is prone to complications involving the cardiovascular and nervous systems, as well as the disorders of glucose caused by diabetes, the challenges in anesthetic management for diabetic patients are increased. Regional anesthesia is extensively utilized to block nerve conduction, finding application in both surgical anesthesia and the management of acute and chronic pain, with documented benefits in promoting the restoration of specific aspects of postoperative function^4,5^. Ropivacaine is a local anesthetic commonly used in clinical practice, and continuous nerve block with ropivacaine offers low cardiovascular and central nervous system toxicity, precise anesthetic effects, and prolonged analgesia^6^. Encouragingly, it has recently become a popular anesthetic option in the perioperative management of patients with type 1 and type 2 diabetes mellitus, as regional anesthesia offers superior postoperative analgesia compared to general anesthesia while avoiding the latter’s cardiopulmonary and insulin-resistance effects^7^.

Despite the well-recognized advantages of regional anaesthesia, it is important to note that once diabetes has progressed to affect the peripheral nervous system, the use of local anaesthetics can lead to a range of pathophysiological consequences. Notably, diabetic peripheral neuropathy represents the most common complication of both type 1 and type 2 diabetes^8^. Diabetic neuropathic nerves are more difficult to stimulate^9^, more sensitive to local anesthetics^10^, and potentially more susceptible to local anesthetic-induced neurotoxicity^11^. Clinically, previous work has shown that when comparing the clinical effects of regional anesthesia between patients with diabetic neuropathy and healthy controls, the analgesic effect in neuropathic patients was prolonged by 50% to 100%, and the blocks exhibited a faster onset time^12^. Navs are essential for the initiation and propagation of action potentials, thereby underpinning electrical signaling that plays a key role in nociception^13^. Diabetic neuropathic nerves exhibit altered Navs, rendering them more sensitive to local anesthetics while being more difficult to stimulate^14,15^.

Thus, patients with diabetes who receive prolonged blocks with high concentrations of local anaesthetic agents may be at additional risk of developing pressure ulcers and delayed mobilisation. Hoope et al. demonstrated that rats with type 2 diabetes-induced neuropathy exhibited prolonged nerve block duration compared to control rats, and the ED_50_ of lidocaine for motor block in diabetic rats was 64% of that in control animals^10^. In addition, a prospective study demonstrated that, in the context of ropivacaine-induced blocks, the durations of sensory and motor blocks are significantly prolonged in patients with type 2 diabetes compared with non-diabetic patients^16^. Accordingly, selecting an appropriate drug concentration is critical for mitigating the risk of hospital-acquired complications. In this study, we investigated the ED_50_ of ropivacaine in diabetic rats and the potential mechanisms governing its effects.

First, we determined the ED_50_ of ropivacaine using the Dixon up-and-down method in normal and diabetic rats. Second, we observed that the altered Navs may impact the ED_50_. Finally, ropivacaine administered at its ED_50_ did not exacerbate peripheral nerve injury in diabetic rats. These findings provide a fundamental basis for subsequent in-depth research on the specific regulatory mechanisms of Navs on the ED_50_ of ropivacaine, as well as for its clinical application.

## Results

### The ED_50_ of ropivacaine was identified in diabetic and normal rats

**Fig. 1:**
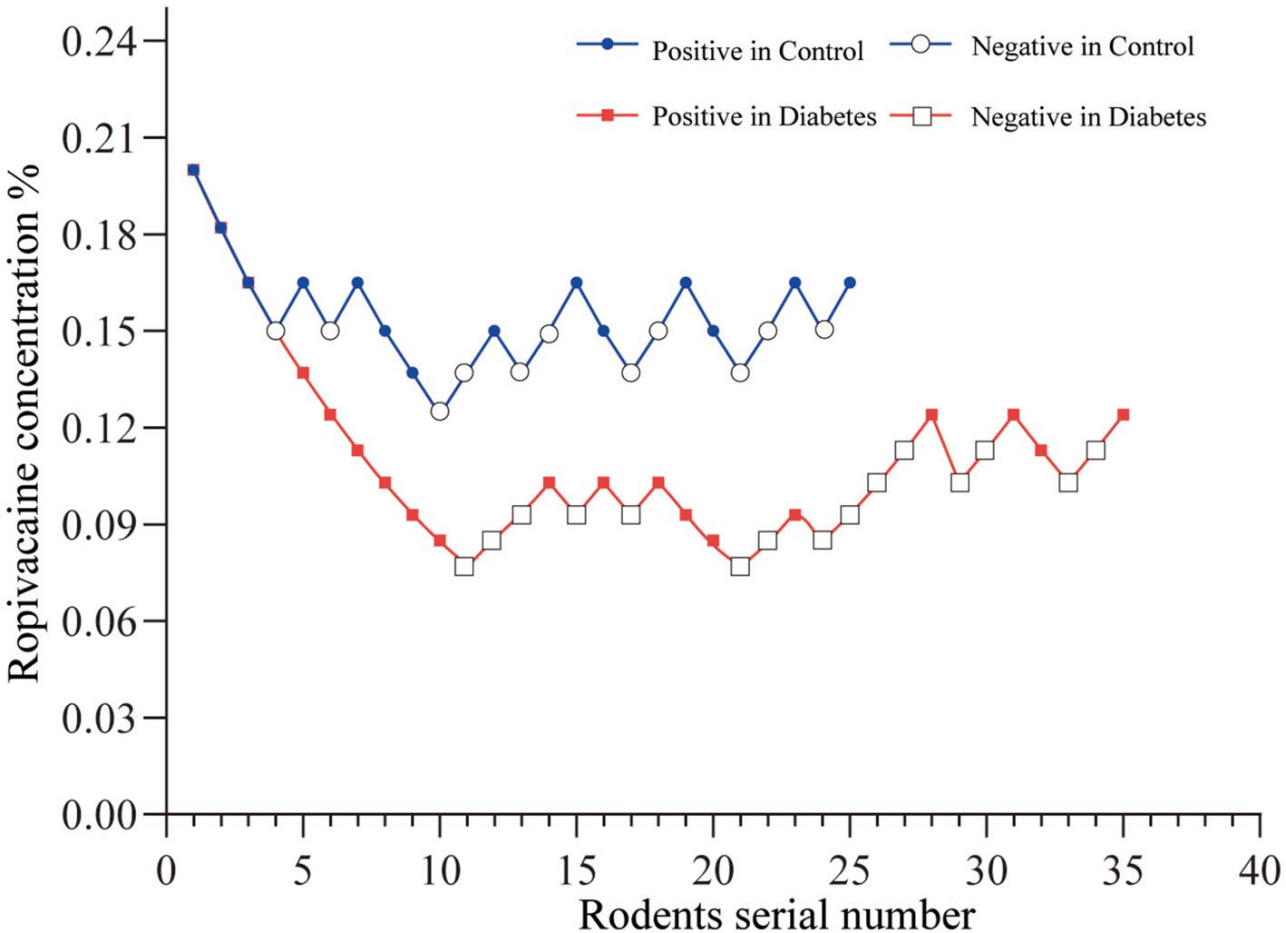
In vivo ED_50_ of ropivacaine in sciatic nerve block. ED_50_ was investigated in Diabetic and Control rats according to the Dixon up-and-down method. A total of 35 diabetic and 25 control rats were necessary to determine the ED_50_. “Positive response in control/diabetic rats” indicates that the rat achieved effective sciatic nerve motor block after ropivacaine administration, defined as a score of 3 based on the 0–3 motor response and pain-related behavior grading system; “Negative in control/diabetes” indicates that effective motor block was not achieved (motor response score < 3). These positive/negative outcomes were sequentially recorded to guide dose gradient adjustments (adjacent concentrations maintained a 1:1.1 ratio) in the Dixon up-and-down method, with seven cross-reactions set as the termination criterion, ultimately supporting the calculation of ED_50_ and its 95% CI.

ED_50_ is a fundamental quantitative index in pharmacology, toxicology, and medical research. As shown in Fig. 1, the Dixon up-and-down method was employed to determine the ED_50_ of ropivacaine for sciatic nerve motor block in both diabetic and control rats^17^. The ED_50_ value in diabetic rats was 0.100% (95% CI 0.043–0.119), which was significantly lower than that in control rats (0.142%; 95% CI 0.123–0.162). Sequential dose–response analysis revealed a consistent pattern of heightened sensitivity in diabetic animals, with fewer subjects achieving effective motor block at equivalent concentrations. These findings indicate that diabetes mellitus enhances peripheral nerve susceptibility to ropivacaine-induced conduction blockade, likely attributable to underlying neuropathic alterations.

### The sciatic nerve of diabetic rats exhibited changes in MNCV and histology

**Fig. 2:**
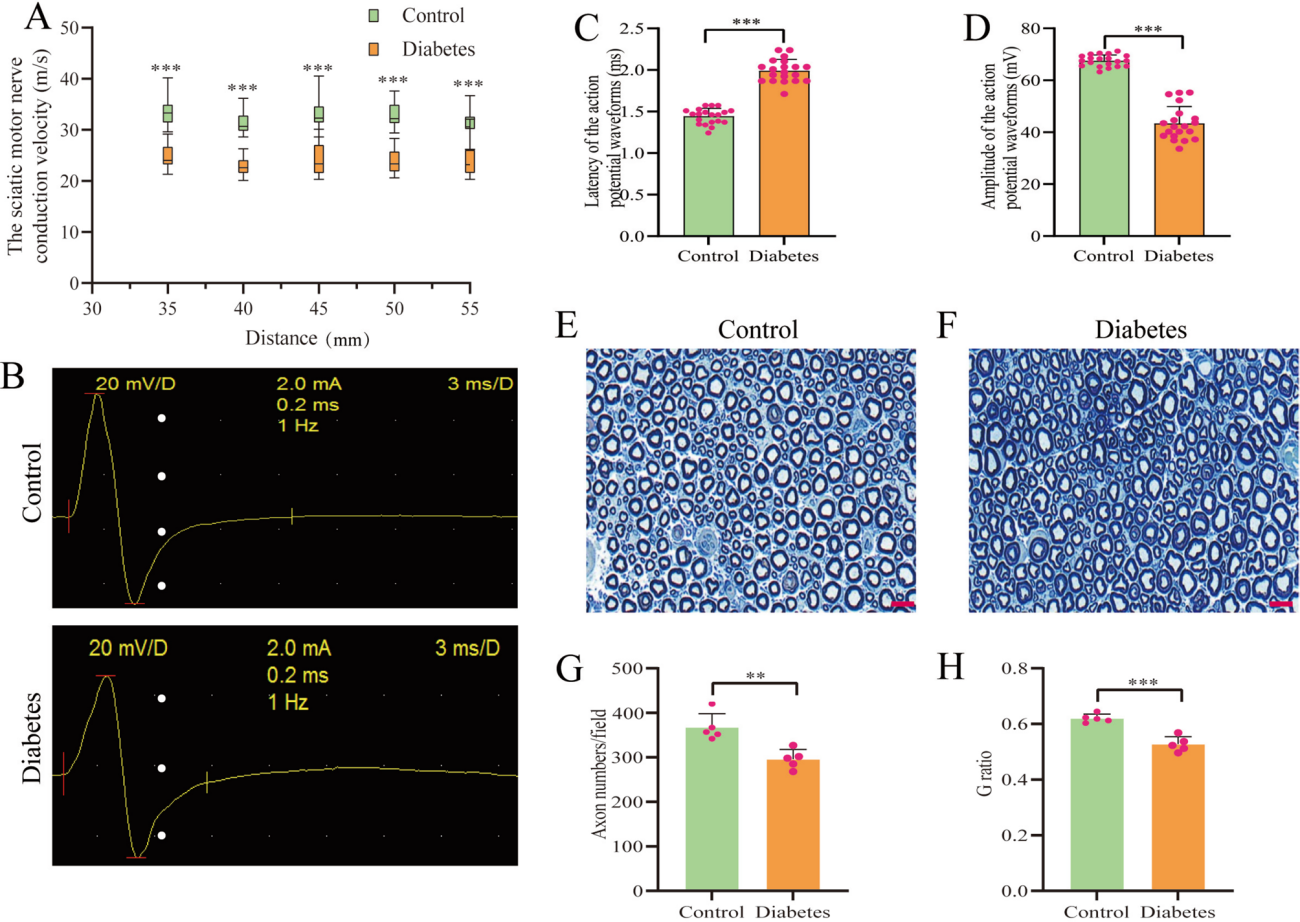
The motor nerve conduction velocity, axon density and G-ratio of sciatic nerve. (**A**) Comparison of sciatic nerve conduction velocity at different segments between Diabetes and Control groups (*n* = 20). (**B**) Demonstration of the action potential waveform diagram in two groups of rats (*n* = 5). (**C**) The latency of the action potential waveform in two groups. (**D**) The amplitude of the action potential waveform in Diabetes and Control groups. (**E**) Toluidine blue staining of rat sciatic nerves in Control rats. Scale bar: 0.01 mm. (**F**) Toluidine blue staining of rat sciatic nerves in Diabetic rats. Scale bar: 0.01 mm. (**G**) Axon density (number of axons per field) in Diabetes and Control groups. (**H**) G-ratio for axon diameters of 3–4 mm in two groups of rats. Significant differences between mean values were determined by Student’s t-test. Mean ± SD, dots represented individual rats. **p < 0.01, ***p < 0.001 compared to Control group.

To investigate the potential mechanism underlying the reduced ED_50_ in diabetic rats, we observed the MNCV and histological structure of the sciatic nerve^18,19^. Specifically, we measured MNCV across multiple nerve segments and evaluated histological features to identify structural and functional alterations. First, we measured the MNCV of sciatic nerves at different lengths (35 mm, 40 mm, 45 mm, 50 mm and 55 mm), and the results showed that the conduction velocity in diabetic rats was significantly lower than that in the Control group across all length groups (*P* < 0.001)(Fig. 2A). This significant reduction in MNCV across all measured nerve segments indicated impaired axonal function in diabetic rats. Furthermore, the action potential waveforms showed that the action potential waveform of diabetic rats had lower amplitude and prolonged latency, which indicated a decrease in the number of excited axons in the sciatic nerve and reduced MNCV (Fig. 2B–D). Consistent with these findings, electrophysiological recordings further revealed prolonged latency and reduced amplitude of compound action potentials in diabetic nerves, which is associated with axonal loss and demyelination.

Second, we evaluated the changes in the organizational structure of the sciatic nerve, and the results revealed that compared with the Control group, axon density was remarkably decreased in diabetic rats (Fig. 2E–G). The G-ratio of axons was lower in the Diabetes group compared with the Control group (Fig. 2H). Histological analysis using toluidine blue staining further demonstrated these changes, showing a marked decrease in axon density and a lower G-ratio in diabetic rats relative to controls, which reflects axonal atrophy and compromised myelin integrity. These structural and functional deficits collectively suggest that diabetic neuropathy contributes to heightened local anesthetic sensitivity by reducing the safety margin for impulse propagation, and the electrophysiological function and histological changes of the sciatic nerve may lead to a decrease in ED_50_ in diabetic rats.

### The expression of Navs was decreased in the sciatic nerve and DRG of diabetic rats

**Fig. 3:**
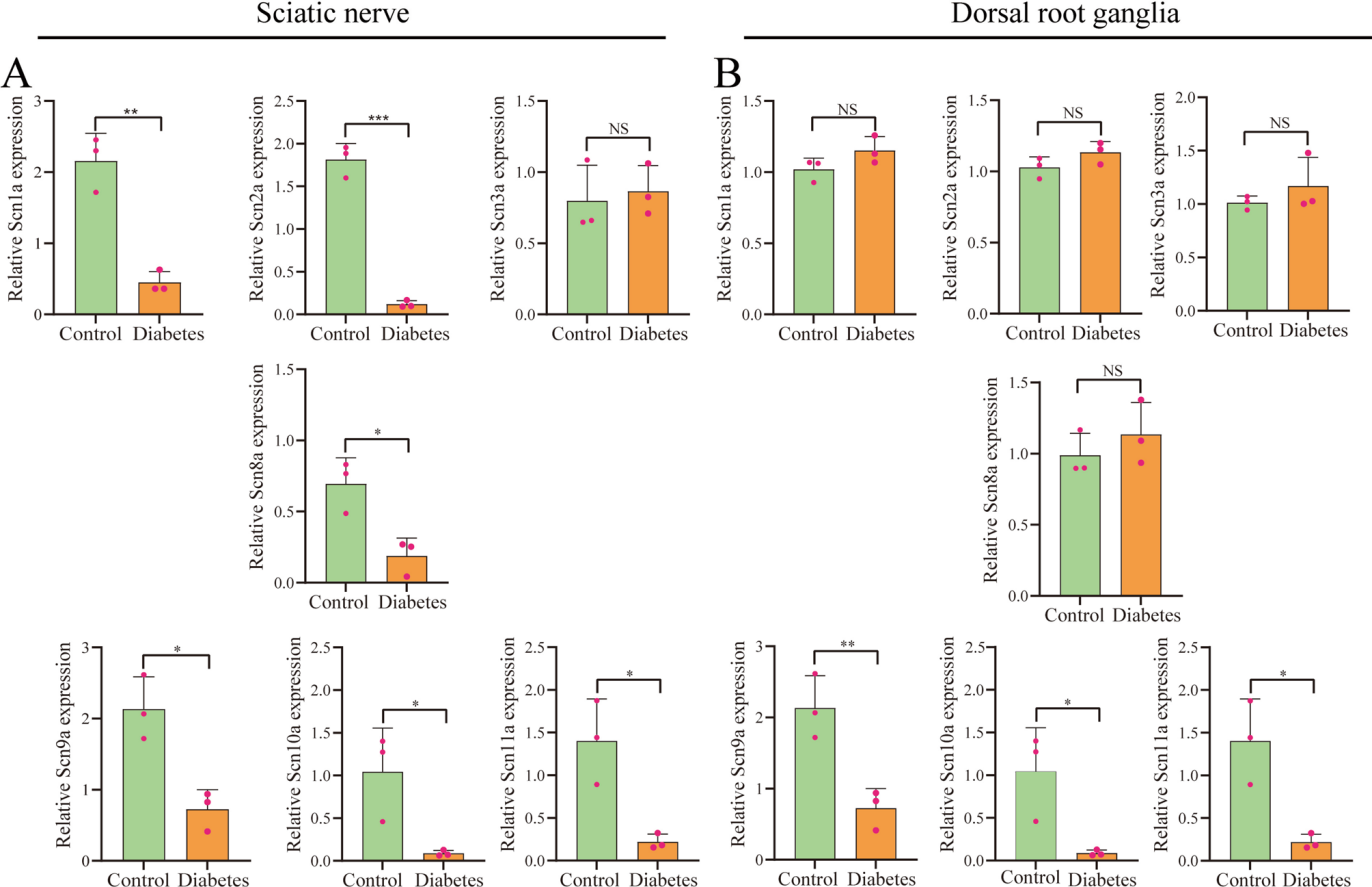
Relative expression of scn genes in sciatic nerve and DRG by RT-PCR. (**A**) RT-PCR analysis of *Scn1a/2a/3a/8a/9a/10a/11a* relative expression in sciatic nerve (Control vs. Diabetes groups). (**B**) RT-PCR Analysis of *Scn1a/2a/3a/8a/9a/10a/11a* relative expression in DRG (Control vs. Diabetes groups). Data presented as mean ± SD. NS: *p* > 0.05, *p < 0.05, **p < 0.01, ***p < 0.001. Dots represented individual rats.

**Fig. 4:**
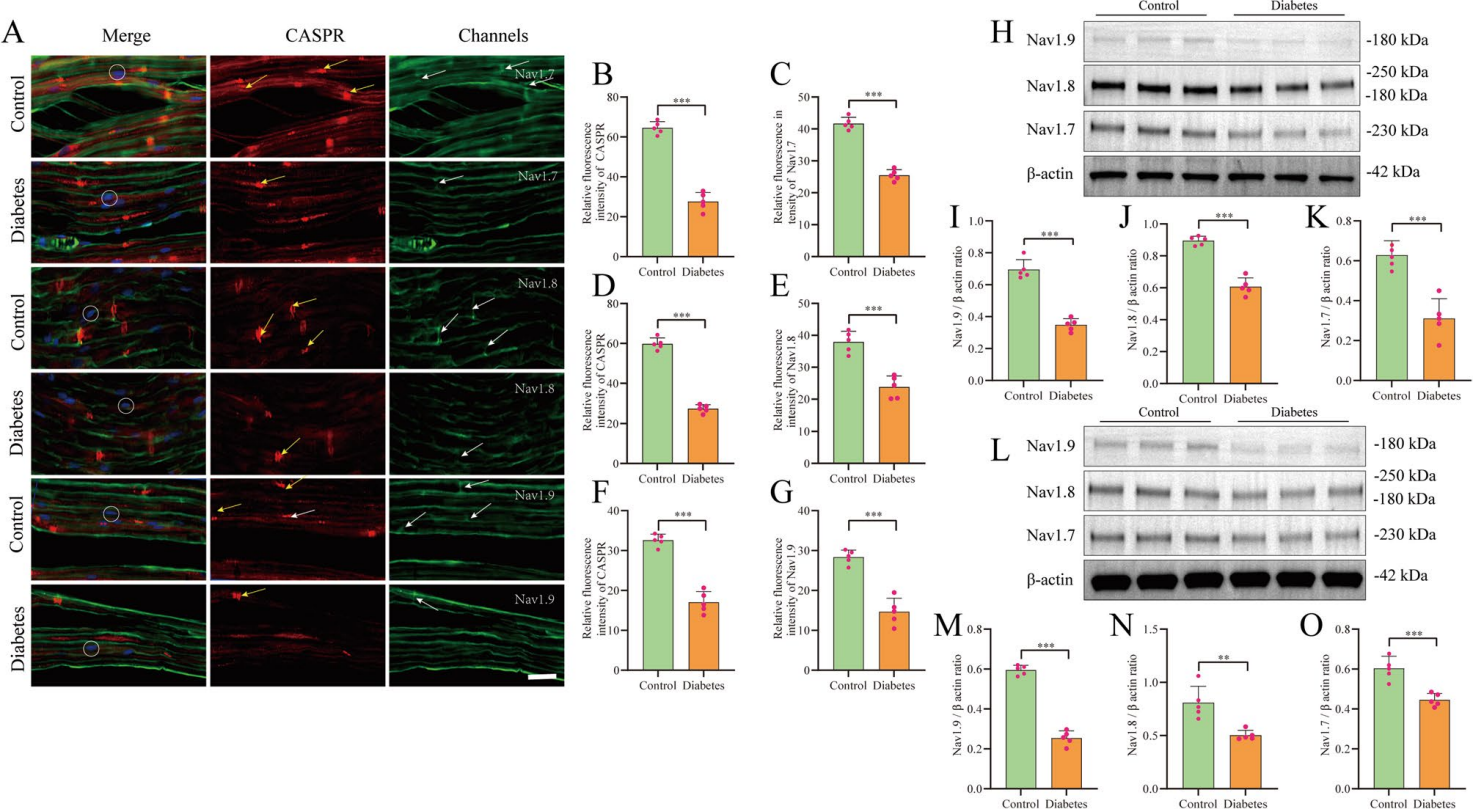
Analysis of Nav1.7, Nav1.8, Nav1.9, and CASPR via immunofluorescence and western blot in Control and Diabetes groups. (**A**) Immunofluorescence merged images of Nav subtypes and CASPR in sciatic nerve. (The white circle indicates DAPI, the yellow arrow indicates CASPR and the white arrow indicates Navs, Scale bar: 50 μm). (**B**,**C**) Representative the relative fluorescence intensity of CASPR and Nav1.7 in Control vs. Diabetes groups. (**D**,**E**) Bar graph displayed the relative fluorescence intensity of CASPR and Nav1.8 in two groups. (**F**,**G**) Representative the relative fluorescence intensity of CASPR and Nav1.9 in Control vs. Diabetes Groups. (**H**) Representative western blot protein bands of Nav subtypes in the sciatic nerve. (**I**–**K**) Quantitative analysis of protein bands of Nav1.7, Nav1.8 and Nav1.9 in Control vs. Diabetes Groups. (**L**) Representative western blot protein bands of Nav subtypes in DRG. (**M**–**O**) Bar graph displayed the quantitative analysis of protein bands of Nav1.7, Nav1.8 and Nav1.9. Significant differences between mean values were determined by Student’s t-test. Data presented as mean ± SD. **p < 0.01, ***p < 0.001. Dots represented individual rats.

Local anesthetics act on sodium ion channels, thereby mediating their blocking effect on nerve signal conduction^20^. To find out if the expression of sodium ion channels in the sciatic nerve and DRG was altered in diabetic rats, we employed Real-Time Polymerase Chain Reaction (RT-PCR), western blot, and immunofluorescence localization to investigate the expression and subcellular localization of ion channels. Our results, as determined by RT-PCR, showed that the expression levels of *Scn1a* (Nav1.1)*, Scn2a* (Nav1.2)*, Scn8a* (Nav1.6)*, Scn9a* (Nav1.7)*, Scn10a* (Nav1.8) *and Scn11a* (Nav1.9) were notably decreased in the sciatic nerve of diabetic rats compared with the normal rats (Fig. 3A). However, only the expression levels of *Scn9a,Scn10a* and *Scn11a* were significantly reduced in DRG (Fig. 3B). As widely reported in previous studies, three voltage-gated sodium channels, namely Nav1.7, Nav1.8, and Nav1.9, are selectively expressed in primary afferent nociceptors^21,22^. Our data collectively demonstrated that the expression levels of Nav1.7, Nav1.8 and Nav1.9 on the nerve fibers were remarkably lower in Diabetes group than that in Control group (Fig. 4A). Moreover, the expression levels of the three types of ion channel proteins in the sciatic nerve and DRG also showed consistent results with those mentioned above (Fig. 4H,L). Taken together, we found that the expression levels of sodium ion channels were decreased in the sciatic nerve and DRG of diabetic rats, which may affect the ED_50_ of ropivacaine.

CASPR is primarily localized to the cell membrane of neurons, and is particularly highly enriched in the paranodal region. By binding to contactin (a protein on the surface of adjacent cells), CASPR forms a stable protein complex, which in turn ensures the normal propagation of action potentials^23^. Consequently, it is frequently used in studies related to sciatic nerve myelin repair^24^. In this study, the results demonstrated that the expression level of CASPR was significantly reduced in the sciatic nerve of diabetic rats (Fig. 4A). Given the critical role of CASPR in maintaining paranodal junction stability and myelin-axon integrity^25^, this reduction strongly indicated potential abnormalities in the structural integrity, myelin function, and neural signal conduction of the sciatic nerve. The above results are consistent with the abnormal histological changes in sciatic nerve and the decrease in motor conduction velocity.

### The ED_50_ of ropivacaine did not impair the MNCV or induce histological abnormalities in the sciatic nerve

**Fig. 5:**
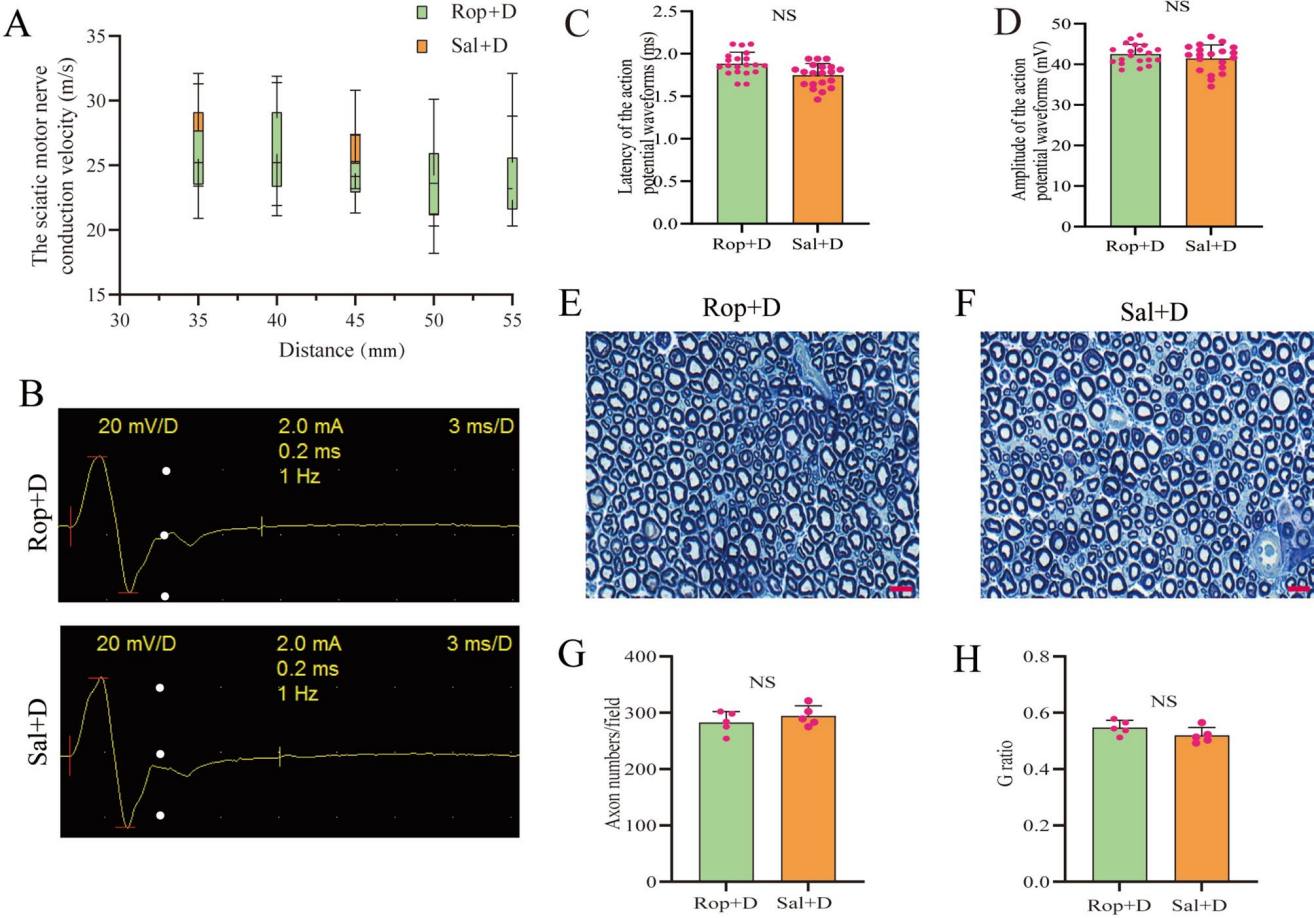
Ropivacaine at ED_50_ dose did not impair the motor nerve conduction velocity, axon density and G-ratio of sciatic nerve. (**A**) Representative the MNCV of sciatic nerve at different segments in diabetic rats treated with or without ropivacaine (*n* = 15). (**B**) Demonstration of the action potential waveform diagram in Rop + D group and Sal + D group rats (*n* = 5). (**C**) The latency of the action potential waveform in two groups. (**D**) The amplitude of the action potential waveform in in Rop + D and Sal + D group rats. (**E**) Toluidine blue staining of rat sciatic nerves in diabetic rats treated with ropivacaine. Scale bar: 0.01 mm. (**F**) Toluidine blue staining of rat sciatic nerves in diabetic rats treated without ropivacaine. Scale bar: 0.01 mm. (**G**) Axon density (number of axons per field) in two groups of rats. (**H**) G-ratio for axon diameters of 3–4 mm in two groups. Significant differences between mean values were determined by Student’s t-test. Mean ± SD, dots represented individual rats. NS: *p* > 0.05 compared to Sal + D group.

To evaluate whether administration of ropivacaine at its ED_50_ exacerbates nerve injury under diabetic conditions, and to further determine whether this dose causes sciatic nerve damage in diabetic rats, we designed a comparative study to assess sciatic nerve conduction velocity, electrophysiological parameters, and histological structure in ropivacaine-treated diabetic (Rop + D) and saline-treated diabetic group (Sal + D). This study aimed to evaluate the safety of ropivacaine at its reduced ED_50_ dose in diabetic peripheral nerves and to provide experimental evidence supporting its clinical application in this vulnerable population. Seven days post-sciatic nerve block, we first measured MNCV across all segments of the sciatic nerve. The results showed no significant differences in MNCV values between the Rop + D and Sal + D groups (Fig. 5A), indicating that ED_50_-dose ropivacaine did not impair the conduction function of diabetic sciatic nerves.

To further verify electrophysiological integrity, we performed action potential waveform analysis. The waveform plots revealed similar patterns between the two groups, and no significant differences were observed in the latency or amplitude of action potentials (Fig. 5B–D). These electrophysiological findings collectively suggest that ED_50_-dose ropivacaine does not induce additional electrophysiological dysfunction in diabetic sciatic nerves, consistent with the MNCV results. In addition to functional assessments, we examined the histological structure of the sciatic nerve to evaluate whether ED_50_-dose ropivacaine causes structural damage to diabetic peripheral nerves. Specifically, we analyzed axon density and the G-ratio, a key indicator that reflects myelin sheath thickness relative to axon diameter.. Histological examination confirmed no significant differences in either axon density or G-ratio between the Rop + D and Sal + D groups (Fig. 5G,H), indicating that ropivacaine exposure at this dose did not result in structural abnormalities such as axon loss or myelin sheath damage in diabetic sciatic nerves.

Collectively, these findings demonstrate that the ED_50_ of ropivacaine does not impair MNCV, induce electrophysiological dysfunction, or cause histological abnormalities in the sciatic nerves of diabetic rats. Importantly, the results suggest that this reduced ED_50_ dose may not be associated with exacerbated neurotoxicity in diabetic peripheral nerves, supporting its safe use in diabetic patients requiring regional anesthesia.

### Ropivacaine at ED_50_ dose did not significantly alter Navs expression

**Fig. 6:**
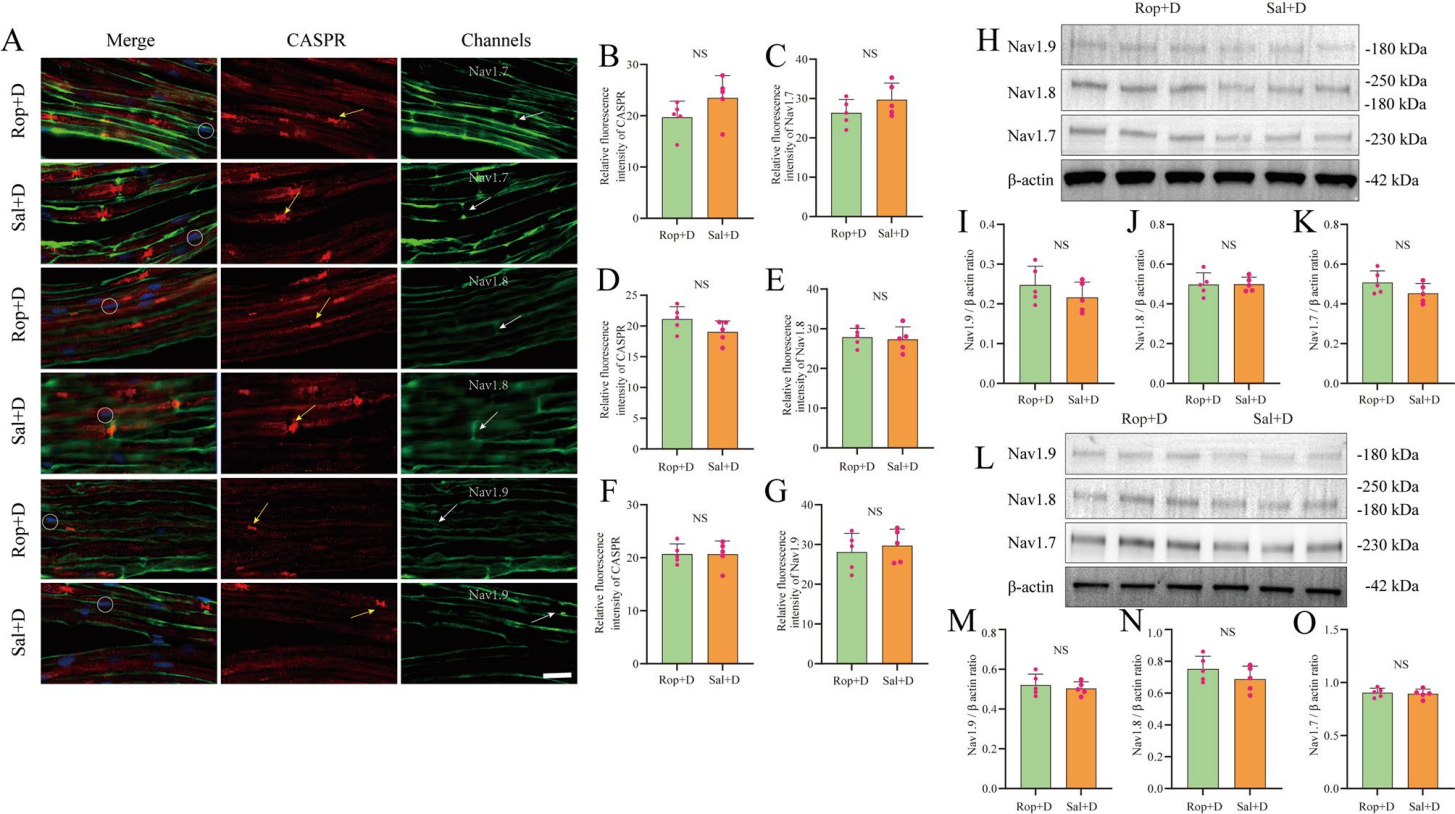
Ropivacaine at ED_50_ dose did not notably alter Navs expression. (**A**) Merged immunofluorescence images showing Nav subtypes and CASPR in the sciatic nerve. White circles: DAPI; Yellow arrows: CASPR; white arrows: Nav subtypes. Scale bar: 50 μm. (**B**,**C**) Representative images showing the relative fluorescence intensity of CASPR and Nav1.7 in Rop + D group and Sal + D group. (**D**,**E**) Bar graphs summarizing the relative fluorescence intensity of CASPR and Nav1.8 in the two groups. (**F**,**G**) Representative images illustrating the relative fluorescence intensity of CASPR and Nav1.9 in the Rop + D and Sal + D groups. (**H**) Representative western blot images of Nav channel subtypes in the sciatic nerve. (**I**–**K**) Quantitative analysis of Nav1.7, Nav1.8, and Nav1.9 protein expression levels in diabetic rats treated with and without ropivacaine. (**L**) Representative western blot images of Nav channel subtypes in the DRG. (**M**–**O**) Bar graphs showing the quantitative analysis of Nav1.7, Nav1.8, and Nav1.9 protein expression. Data are presented as mean ± SD; individual data points represent values from each rat. Significant differences between groups were determined by Student’s t-test: NS: *p* > 0.05.

Next, we assessed whether the expression levels of Nav subtypes and CASPR were altered in diabetic rats following ropivacaine treatment. Specifically, we investigated whether administration of ropivacaine at its ED_50_ dose modulates the expression of Navs and the paranodal protein CASPR in diabetic rats. Immunofluorescence staining revealed no significant differences in the fluorescence intensity of Nav1.7, Nav1.8, or Nav1.9 in sciatic nerve fibers between the Rop + D and the Sal + D group, consistent with our observation that no significant changes in Nav subtype expression were detected via immunofluorescence (Fig. 6A). Similarly, Western blot analysis of sciatic nerve and DRG tissues showed no statistically significant changes in the protein levels of these Nav subtypes following ropivacaine treatment. In the sciatic nerve and DRG of ropivacaine-treated rats, a slight upward trend in the protein expression of Nav1.7, Nav1.8, and Nav1.9 was observed; however, this trend did not reach statistical significance (Fig. 6H, L), mirroring the nonsignificant increase noted in the Rop + D group. CASPR expression also remained unchanged, indicating that the structural integrity of paranodal regions was not adversely affected, which aligns with the absence of significant changes in CASPR expression levels in nerve fibers.

Taken together, these findings suggest that ropivacaine may not markedly alter the expression levels of Nav subtypes and appears to exert no significant effect on sodium channel protein production in the sciatic nerve of diabetic rats, nor does it appear to disrupt nerve structure. These results further imply that ropivacaine at its ED_50_ may not further dysregulate sodium channel expression or disrupt nodal architecture in the context of diabetic neuropathy, possibly supporting its safety profile; however, further studies are needed to confirm these observations and to explore potential subtle effects that may not have reached statistical significance in the current analysis.

## Discussion

Herein, our study demonstrates that STZ-induced diabetic rats exhibit a significantly reduced ED_50_ of ropivacaine for motor blockade, indicating enhanced sensitivity to local anesthetics. Our data further reveal that these changes are associated with structural and functional alterations in the sciatic nerve, including decreased MNCV, reduced axon density, and diminished expression levels of key Nav subtypes and the paranodal protein CASPR. Crucially, administration of ropivacaine at the ED_50_ dose did not exacerbate neurophysiological or histological damage in diabetic rats, suggesting a favorable safety profile under these conditions.

The observed reduction in ropivacaine ED_50_ aligns with a growing body of evidence indicating heightened local anesthetic sensitivity in diabetic neuropathy. Our findings corroborate the work of Ten Hoope et al., who reported a 64% decrease in the ED_50_ of lidocaine in type 2 diabetic rats, and the clinical observations of Cuvillon et al., who documented prolonged ropivacaine-induced blocks in diabetic patients^10,16^. The present study extends these findings by systematically linking this pharmacodynamic shift to specific pathological alterations. The diabetic rats in our model exhibited classic hallmarks of advanced neuropathy, including slowed MNCV, diminished action potential amplitude, and histological evidence of reduced axon density and G-ratio, indicative of axonal degeneration and compromised myelin integrity.

A central mechanistic insight from our work is the significant downregulation of multiple Nav subtypes, specifically Nav1.7, Nav1.8, and Nav1.9, in the sciatic nerve and DRG of diabetic rats. These channels are pivotal for action potential initiation and propagation in nociceptive and motor neurons^26,27^. While some studies have reported upregulation of certain Nav1.3 or Nav1.7 in early, painful stages of neuropathy^28,29^, our data from a model of established diabetes support a paradigm where chronic neuropathic progression leads to a generalized decrease in Nav expression (our model used 5 weeks of diabetes). This decline may stem from neuronal degeneration or represent a compensatory downregulation to mitigate hyperexcitability. From a pharmacodynamic perspective, a reduced complement of functional sodium channels would lower the threshold for conduction blockade^30^, as fewer channels need to be occupied by ropivacaine to achieve failure of impulse propagation. This provides a plausible molecular explanation for the reduced ED_50_. In addition to Nav alterations, we identified a significant reduction in the expression of CASPR, a critical component of the paranodal junction that ensures axo-glial adhesion and efficient saltatory conduction^31,32^. The coordinated downregulation of CASPR and Nav points to a widespread disruption of nodal and paranodal architecture in diabetic nerves. The loss of CASPR, known to destabilize the node of ranvier and slow conduction velocity^33^, aligns perfectly with our electrophysiological findings. This structural compromise likely synergizes with the reduced Navs availability to enhance nerve susceptibility to local anesthetics, as a damaged myelin sheath may facilitate drug penetration and a diminished conduction reserve lowers the safety factor for blockade^34^. Thus, the loss of CASPR and subsequent paranodal disruption likely contributes to heightened ropivacaine sensitivity through a dual mechanism:^1^ facilitating drug diffusion to the axonal membrane by compromising the myelin barrier, and^2^ reducing the safety margin for impulse propagation due to impaired saltatory conduction. Our data do not distinguish the relative contribution of each, but both are plausible and not mutually exclusive.

A key question regarding the observed Nav downregulation is whether it represents a primary response to diabetic metabolic dysfunction or a secondary effect of axonal density reduction and atrophy. Our data support a dual mechanism, where downregulation of Nav1.7/1.8/1.9 is primarily driven by diabetes-induced metabolic stress (e.g., advanced glycation end products accumulation and oxidative stress), and axonal structural damage exerts a secondary amplifying effect by reducing the membrane surface area for channel localization and disrupting neurotrophic support. Temporally, Navs dysregulation was detected at 5 weeks post-diabetes induction, concurrent with early axonal changes but prior to overt axonal degeneration. This observation is consistent with previous reports of sodium channel alterations preceding structural nerve damage in early diabetic peripheral neuropathy^14,15^. Molecularly, hyperglycemia-induced advanced glycation end products accumulation, oxidative stress, and impaired peroxisome proliferator-activated receptor γ signaling directly regulate Navs transcription^28,29^. This is evidenced by reduced *Scn9a/10a/11a* mRNA levels in the sciatic nerve and DRG, a finding incompatible with mere structural loss of axons. Meanwhile, axonal density reduction and atrophy likely exacerbate Navs downregulation by decreasing the membrane surface area available for channel localization and disrupting retrograde neurotrophic support^13,26^, creating a vicious cycle that amplifies neuropathic dysfunction. The coordinated downregulation of Navs and CASPR, a paranodal protein regulated by hyperglycemia-disrupted axo-glial signaling^31,33^, further validates that both molecular and structural changes are driven by common metabolic insults, with structural damage secondarily amplifying Navs dysregulation. This pathophysiological hierarchy explains why Navs downregulation correlates with reduced axon density in our model and underscores the importance of targeting metabolic pathways to mitigate Navs-mediated anesthetic sensitivity in diabetic patients.

From a clinical standpoint, it remains unclear whether heightened sensitivity in diabetic nerves also increases vulnerability to local anesthetic-induced neurotoxicity^35^. These findings suggest that administration of ropivacaine at the ED_50_ dose (0.100%) does not induce additional functional or structural neural damage in diabetic rats within the 7-day observation period. No significant differences in MNCV, axon density, G-ratio, or Navs/CASPR expression were observed between ropivacaine-treated and saline-treated diabetic animals. This is a clinically reassuring finding, as it suggests that reduced doses of ropivacaine, which are tailored to the heightened sensitivity of diabetic patients, may not increase the risk of iatrogenic nerve injury when used in these patients. Diabetic patients, who often require prolonged postoperative analgesia, face a dilemma: under-dosing leads to inadequate pain relief, while over-dosing or using standard concentrations risks prolonged motor block (potentially leading to pressure sores and delayed mobilization) and neurotoxicity. Our data suggest that titrating ropivacaine concentrations downward in diabetic patients, in recognition of their altered pharmacodynamics, can achieve effective blockade without incurring additional neurotoxic risk. Furthermore, this supports the cautious use of ropivacaine alone at lower doses, potentially circumventing the need for adjuvants like dexmedetomidine, which has been shown to exacerbate ropivacaine-induced nerve injury in diabetic states^18^. However, this strategy should be interpreted with caution when considering continuous peripheral nerve blocks. In continuous infusion protocols, the risk of prolonged motor blockade and local anesthetic-induced nerve injury may be higher, particularly in vulnerable diabetic nerves. Therefore, the safety and efficacy of reduced ropivacaine concentrations, with or without adjuvants, in continuous infusions for diabetic patients remain to be determined.

It is important to note, however, that the ED_50_ value obtained in rats (0.100%) is considerably lower than concentrations typically used in clinical practice. This discrepancy is expected and can be attributed to well-established interspecies differences in nerve morphology, metabolism, and pharmacokinetics. Therefore, this specific value should not be directly extrapolated to clinical dosing. Rather, the primary value of our finding lies in the mechanistic insight it provides: it robustly demonstrates why diabetic nerves are more sensitive to local anesthetics. Heightened sensitivity driven by Navs downregulation and structural nerve changes supports a clinical strategy of cautious dose individualization in diabetic patients. Instead of fixed standard concentrations, clinicians should consider initiating blocks at lower concentrations and titrating to effect based on individual response. This approach aligns with the principles of precision medicine, aiming to achieve effective anesthesia while minimizing the risk of prolonged blockade or systemic toxicity in this vulnerable population.

Several limitations of our study warrant consideration. First, the STZ-induced model primarily mimics type 1 or severe insulin-deficient type 2 diabetes mellitus. The generalizability of our findings to the more prevalent form of type 2 diabetes mellitus characterized by insulin resistance requires validation in models such as high-fat diet/STZ-induced rodents, though the core relevance of our results to type 2 diabetes mellitus with diabetic peripheral neuropathy is supported by shared pathophysiology and existing literature. Second, the 7-day post-block observation period is relatively short and insufficient to rule out delayed neurotoxic effects in this vulnerable diabetic population. Moreover, our single-injection model does not replicate the clinical scenario of continuous peripheral nerve blocks or repeated ropivacaine infusions, which may carry different risks of delayed neurotoxicity or cumulative nerve injury. Future studies using prolonged or continuous administration protocols are warranted to evaluate the long-term safety of reduced-dose ropivacaine in diabetic neuropathy. Third, while our study demonstrates a strong correlation between reduced Nav expression and decreased ropivacaine ED_50_ in diabetic rats, it does not establish direct causality. Future studies employing targeted knockdown or inhibition of specific Nav subtypes in healthy animals would be necessary to confirm that Nav downregulation itself is sufficient to enhance local anesthetic sensitivity. Finally, this study was conducted exclusively in male rats. Given known sex differences in pain biology and drug responses, our findings may not fully apply to females, highlighting the need for future research to investigate potential sex-dependent variations in ropivacaine sensitivity and Nav subtype expression in diabetic neuropathy.

In conclusion, this study elucidates that the reduced ED_50_ of ropivacaine in diabetes is underpinned by a complex pathophysiology involving Nav downregulation and CASPR-associated structural disintegration of the nerve. Importantly, leveraging this heightened sensitivity by using a lower, effective dose of ropivacaine appears to be a safe strategy that does not compound the underlying nerve injury. These findings support the clinical practice of using lower local anesthetic concentrations in diabetic patients to achieve effective analgesia while minimizing potential toxicity. Further research is warranted to elucidate the precise signaling pathways governing Nav expression in diabetes and to develop targeted strategies for optimizing regional anesthesia in this vulnerable population.

## Experimental procedures

### Animals

All experimental procedures and protocols used in this study were approved by the Animal Ethics Committee of the Hebei Medical University Third Hospital (Permit No. Z 2023–022-1). The study was designed and reported in strict accordance with the ARRIVE guidelines (https://arriveguidelines.org). Adult male Sprague–Dawley rats (Department of Laboratory Animal Science, Hebei Medical University; No.SCXKJI 2022–001), weighing 210 ~ 260 g, were housed in an animal care facility thermostatically maintained at 22 ± 2 °C with a 12-h light–dark cycle. Animals were acquired at 14 weeks of age and allowed a 6-week acclimatization period with free access to water and food. Animals were then divided into distinct groups in accordance with the experimental design. We confirm that all methods in this study were performed in accordance with the relevant guidelines, specifically the Guidelines of the American Veterinary Medical Association. Rats were euthanized using a chemical method via inhalational anesthesia: 7% sevoflurane was administered in a transparent anesthetic chamber, inducing rapid unconsciousness, followed by irreversible respiratory and cardiac arrest to ensure humane euthanasia.

### Diabetic rats model

As expected, following previous reports^36^, the rats were fasted overnight and then given an intraperitoneal injection of freshly prepared streptozotocin (STZ, 55 mg/kg, Cayman) in saline (freshly dissolved in 0.05 M, pH 4.0, citrate buffer). Control rats received an injection of saline (the same volume as the STZ solution) instead. Food was returned to the cages 1 h after the injections. Three weeks post-injection, diabetic rats were identified via blood glucose concentration measurements using samples collected from the tail vein. In addition to blood glucose measurements, rats were also evaluated for typical diabetic symptoms (polydipsia, polyphagia, polyuria) weekly. Only rats that maintained blood glucose > 350 mg/dl and exhibited consistent diabetic symptoms for at least 2 consecutive weeks were classified as diabetic^18^. Rats were randomly divided into two groups: the diabetic rats group (Diabetes group) and the control rats group (Control group).

### Sciatic nerve block

Sciatic nerve blockade was performed at the 5th week after induction of diabetes mellitus as described^37^. Animals were briefly anesthetised with 1.5 ~ 3.0% sevoflurane in oxygen by mask and positioned laterally on the operating table with the affected side uppermost. After hair removal, the surgical field was disinfected with iodophor followed by alcohol, then draped with a sterile fenestrated towel. Local infiltration anesthesia was administered via intradermal injection of 0.5% lidocaine. A lateral incision was made in the thigh, and the muscle fascia was bluntly dissected to expose the sciatic nerve. Ropivacaine was injected around the sciatic nerve (proximal to its bifurcation) through the transparent fascia using a 30 G (0.25 mm) disposable insulin syringe. The transparent fascia allowed direct visual confirmation that the nerve was fully enveloped by the injectate with no perineural extravasation in all rats immediately after injection. For precise perineural drug localization and to prevent pooling and leakage, the needle was carefully advanced beneath the fascia, injection was administered at a controlled speed of 0.05 ml/min, and the injection site was gently pressed for 30 s post-injection.

After recovery from general anaesthesia, the sensory response was evaluated first by observing foot withdrawal^38^. The skin fold over the lateral metatarsal bone of the blocked hind paw (for superficial pain) and the tip of the fifth phalanx (for deep pain) was clamped. Assessment was based on a 0–3 grading system for withdrawal reflex and vocalization: 3 = complete block, with no withdrawal response or vocalization. Motor response was then evaluated by observing the toe spreading reflex, which is a vestibular reflex induced by lifting the rat and producing a response with the toes extended and spread^39^. The rat was lifted by the tail to allow its hind limbs to hang freely, and the ability of the hind paw on the blocked side to extend and flex its five toes was evaluated using a 0–3 scoring system:3 = no ability to flex or extend, with dragging.

### The median effective dose of ropivacaine

To assess the ropivacaine ED_50_ in Diabetes group and Control group rats, we performed the Dixon up-and-down method^40^. Specifically, after reviewing different doses used in previous reports^18^, we set the starting concentration at 0.5%. When a successful block was observed, the next rat received the next lower concentration; otherwise, the next higher concentration was administered, with adjacent concentrations maintaining a 1:1.1 ratio. To enhance accuracy, based on preliminary experimental results, seven cross-reactions were established as the termination criterion. Ropivacaine, prepared in various dilutions with 0.9% normal saline to a total volume of 0.2 ml, was injected into the perineural space beneath the clear fascia covering the sciatic nerve using a 30 G needle attached to a tuberculin syringe.

For sciatic nerve block using ropivacaine (ED_50_ in group Diabetes) in diabetic rats, the specific protocol was as follows: the procedure was performed in accordance with the aforementioned sciatic nerve block method, with ropivacaine administered to either the left or right sciatic nerve (Rop + D group) and an equal volume of normal saline administered to the contralateral sciatic nerve (Sal + D group). The experimental manipulation of the rats was carried out 7 days thereafter.

### Measurement of MNCV

After rats were fasted for 12 h, they were briefly anesthetised with 1.5 ~ 3.0% sevoflurane and positioned laterally on the operating table with the affected side uppermost. The subsequent procedures were conducted following the same protocol as the sciatic nerve block. We exposed the entire sciatic nerve, extending from the sciatic notch superiorly to the ankles inferiorly. The sciatic nerve was gently dissected to avoid damage. Immediately after separation, it was maintained at 37 °C and kept moist with warm normal saline throughout the measurement process to prevent tissue dehydration-induced alterations in conduction velocity. The test was initiated promptly thereafter. Electrical stimuli were produced using an electrophysiological monitor (NCC Electrophysiology, China) driven by a positive voltage output at 1 Hz with single twitch stimulation, a 3.0 ms delay, 0.2 ms wave width, and 20 mV intensity. A stimulation needle electrode was positioned at the exit site of the sciatic nerve-located between the femoral tuberosity and ischial tuberosity. The record electrode was inserted at the muscle between the first and second plantar pad of the hind limb^19^. The distance between the stimulation electrode and the recording electrode was measured and recorded. Motor nerve conduction velocity was calculated by dividing the distance between stimulation sites by the difference in latency of the evoked motor response. Electrical stimulation was repeated three times, with the results averaged.

### Harvest the sciatic nerve and DRG

Rats in the Diabetes group, Control group, as well as those in the Rop + D and Sal + D groups at 7 days after sciatic nerve block were anesthetised with 3% sevoflurane, and the sciatic nerve was harvested. The procedure was performed as follows: gently expose a 20-mm segment of the sciatic nerve from the sciatic notch to the mid-thigh, and then excise it and set it aside for subsequent use. After euthanasia with 7% sevoflurane, the rats were placed prone on ice, and DRG were dissected and harvested as described^41^.The midline dorsal hair was shaved and prepped; post-disinfection, a posterior midline longitudinal incision was made from tail to chest. Fascia and muscles over and alongside the spinal column were dissected to expose vertebrae. Muscles covering the lumbar vertebrae were removed, and dissection was continued by sliding down both sides of the lumbar vertebrae until bony prominences (marking the 6th lumbar segment) were palpated. The lumbar spine was excised (upper end: 2 segments below ribs; lower end: including sixth lumbar vertebra) and transferred to ice. Spinous processes and vertebral laminae were removed with rongeurs to expose the spinal cord. Retracting the cord revealed round/oval pale yellow swellings in bilateral intervertebral foramina, identified as DRG.

### RT-PCR

RT-PCR was conducted to examine the expression profiles of various Nav subtypes in the obtained sciatic nerves and DRG samples. The samples were ground in liquid nitrogen, and 200 mg of the ground sample was added to 1 ml of Trizol. After vortexing and 10 min incubation, 0.2 ml of chloroform was added, mixed for 30 s, and incubated on ice for 3 min. Following centrifugation at 12,000 rpm for 15 min at 4 °C, the aqueous phase was transferred to a new tube. RNA was precipitated with 0.5 ml of isopropanol, incubated on ice for 10 min, and centrifuged again. The pellet was washed with 1 ml of ice-cold 75% ethanol, centrifuged, air-dried, and dissolved in DEPC-treated water. 1 μl of RNA was used to assess its quality, concentration, and integrity via a UV spectrophotometer. Reverse transcription was performed using an RT kit following the manufacturer’s instructions (Sango Biotech, Shanghai China). All reactions involved initial denaturation at 95 °C for 15 min; followed by 40 cycles (denaturation at 95 °C for 10 s, annealing at 56 °C for 30 s, and extension at 72 °C for 30 s) using a Heal Force X960 Real-Time PCR Machine (Heal Force, Shanghai, China). Details of the gene primer sequences can be found in Supplementary File 1.

### Toluidine blue staining

For toluidine blue staining, five rats per group were randomly selected. Sciatic nerves were harvested as described in Sect. 2.2, immediately dissected, and fixed in pre‑chilled 2.5% glutaraldehyde‑4% paraformaldehyde at 4 °C for 24 h. After three PBS washes of 15 min each, nerves were post‑fixed in 1% osmium tetroxide at 4 °C for 2 h in the dark, followed by another three PBS washes of 15 min each. Samples were dehydrated in a graded ethanol series from 30 to 100%, 15 min per step, and twice in 100% acetone for 15 min each. Infiltration was performed with acetone‑812 resin mixtures at ratios of 3:1, 1:1, and 1:3 for 2 h each, then in pure 812 resin overnight at 4 °C. Tissues were embedded in pure 812 resin, polymerized at 60 °C for 48 h, and transversely sectioned at 1.0 μm using an ultramicrotome with a glass knife. Sections were baked at 60 °C for 15 min, stained with toluidine blue for 30 s at room temperature, rinsed three times with deionized water for 3 s each, and air‑dried overnight. After mounting with neutral balsam, images were captured under a light microscope with a 100 × oil immersion lens.

### Western blot

The sciatic nerve and DRG tissues were homogenized with RIPA Lysis Buffer (P0013K, Beyotime, China) on ice for 30 min, and then centrifuged at 10,000 rpm for 10 min at 4 °C. Subsequently, the supernatants were taken, and protein concentrations were determined with Pierce BCA Protein Assay Reagent (P00125, Beyotime, China). 20 μg of protein samples were separated via SDS–polyacrylamide gel electrophoresis and subsequently transferred onto PVDF membranes (GVWP04700, Millipore, USA). The membranes were blocked with 5% non-fat milk in TBST for 1 h at room temperature, followed by overnight incubation with primary antibodies at 4℃. After three consecutive washes with TBST (15 min per wash), the membranes were incubated with secondary antibodies for 1 h at room temperature. Finally, protein bands were visualized using a chemiluminescence imaging system (Tanon-5200, China). In this study, each sample was subjected to two independent western blot replicates, which were performed by trained technicians. Details of the primary and secondary antibodies are provided in Supplementary File 2.

### Immunofluorescent assay

The rats were anesthetized and subjected to cardiac perfusion with PBS, followed by perfusion with ice-cold 4% paraformaldehyde. The sciatic nerves were then harvested, fixed in 4% paraformaldehyde, and transferred to 30% sucrose solution at 4 °C for overnight incubation. Tissues were embedded in optimal cutting temperature compound, frozen, and cryosectioned. Subsequently, 15-μm tissue sections were permeabilized, blocked, and incubated with specific antibodies. Primary antibodies used were of rabbit origin. After incubation with the primary antibodies, the sections were incubated with Alexa Fluor-conjugated secondary antibodies at 37 °C for 1 h. Finally, nuclei were stained for 5 min in the dark with DAPI (Bestbio, China), and then observed under a fluorescence microscope (Leica DM IL LED, USA). All quantitative analyses were conducted using ImageJ/Fiji Software (version 1.54, National Institutes of Health, USA) in a blinded manner. Three images were randomly selected from each animal for analysis. Mean fluorescence intensity (MFI; MFI = sum of integrated optical density/area) was calculated as the ratio of total fluorescence intensity to the total area of the analyzed region, for quantifying the positively stained area. All experimental procedures were performed by a trained professional who was blinded to group assignments. Detailed information on the antibodies used is provided in the Supplementary Materials (see Supplementary File 2).

### Statistical analysis

GraphPad Prism 9.0 (GraphPad software, Inc., USA) and SPSS 25.0 (IBM SPSS Statistics, Inc., USA) were used for statistical analysis. Normally distributed data were presented as mean (SD) and analyzed using Student’s t-test. A *P* value < 0.05 was considered statistically significant.

Previous studies^9,42^ indicated that 19–40 subjects were needed to estimate ED_50_ via the up-down method, with sufficient sample size achieved upon obtaining six sequence reversal pairs. Thus, we estimated 40 subjects per group to observe more than six such pairs. Weighted probit regression was used to fit the dose–response curve using maximum likelihood estimation, with subsequent calculation of the ED_50_ and its confidence interval. The difference of ED_50_ values was analyzed using an unpaired* t* test. The 95%CI for ED_50_ values was estimated using Bootstrap Resampling.

We adopted the same group size as Hoope et al.^9^:10 nerves per measurement time point. This meant that 5 animals were required for bilateral injection at each analysis time point, resulting in a total of 35 diabetic animals and 20 control animals being tested. Assuming a common standard deviation of 20% and 1 lost sample per group, this sample size was sufficient to detect a 30% difference in intraneural concentration between healthy and diabetic animals, with a power of 80% and an α level set at *P* < 0.05.

## Supplementary Information

Supplementary Information 1.

Supplementary Information 2.

Supplementary Information 3.

## Data Availability

The data that support the findings of this research are not publicly available at present but will be shared with researchers who provide a valid scientific rationale for their request. Interested parties should contact the corresponding author at 37000628@hebmu.edu.cn to request access.

## Abbreviations

ED_50_: The median effective dose
Navs: Voltage-gated sodium channels
MNCV: Motor nerve conduction velocity
CASPR: The contactin-associated protein
DRG: Dorsal root ganglia
RT-PCR: Real-Time Polymerase Chain Reaction
