# Supplementary material for: Effects of voltage-gated sodium channels on the median effective dose of ropivacaine in diabetic rats
Source: Sci Rep. 2026 May 9;16:21305. doi: 10.1038/s41598-026-49845-w (PMC13347050; doi:10.1038/s41598-026-49845-w)
Supplement: Supplementary file 1 — Supplementary Information 1. [file 41598_2026_49845_MOESM1_ESM.pdf]

Oligo name	Forward primer (5’ to 3’)	Reverse primer (5’ to 3’)
Scn1a	CACTTCAGGGGCTATCGAGG	TCTTCATGTGAGATTCCCCCG
Scn2a	CCCTGTCTTTGCTTTTCCTCT	CCTCGCGTAAGAAAGTGCTGA
Scn3a	GATGGCAGTGACACGTTGAGT	AGCATGCTGAACAAAGAGTATA
Scn8a	GCTCTACGCACTTTCAGGGT	ATGCTGAGCAGCGACTGATT
Scn9a	CCAAAAAGCCAAAGGGCTCC	GCGTTGACCACTACCCTCAT
Scn10a	GGCAACCTAGTGGTGCTCAA	TTGGACTTGTGGCAACTGCT
Scn11a	TCAATCAGCAGCAGAAAAAGTGTAG	GTGTCCTGATTCCTCGAGCC
Actin	AACACGGCATTGTCACCAAC	TTTTCACGGTTGGCCTTAGG
