# Supplementary material for: Effects of voltage-gated sodium channels on the median effective dose of ropivacaine in diabetic rats
Source: Sci Rep. 2026 May 9;16:21305. doi: 10.1038/s41598-026-49845-w (PMC13347050; doi:10.1038/s41598-026-49845-w)
Supplement: Supplementary file 2 — Supplementary Information 2. [file 41598_2026_49845_MOESM2_ESM.pdf]

**Antibodies**	**Source**	**Technique**	**Identifier**
Nav 1.7	Abcam, UK	1:800	ab323849
Nav 1.8	Abcam, UK	1:800	ab63331
SCN11A	Thermo Fisher Scientific(China), China	1:200	PA5-114378
CASPR	Abcam, UK	1:500	ab34151
β-actin	Thermo Fisher Scientific(China), China	1:400	PA5-90724
Scn9a	Thermo Fisher Scientific(China), China	1:100	ASC-008
Scn10a	Thermo Fisher Scientific(China), China	1:100	MA5-45469
SCN11A	Thermo Fisher Scientific(China), China	1:500	ASC-017-200UL
Goat Anti-Rabbit IgG Alexa Fluor 488	Abways, China	1:300	AB0142
Goat Anti-mouse IgG Alexa Fluor 594	Abways, China	1:300	AB0151
