# Supplementary figures and images for: Effects of voltage-gated sodium channels on the median effective dose of ropivacaine in diabetic rats
Source: Sci Rep. 2026 May 9;16:21305. doi: 10.1038/s41598-026-49845-w (PMC13347050; doi:10.1038/s41598-026-49845-w)

Figure 4H

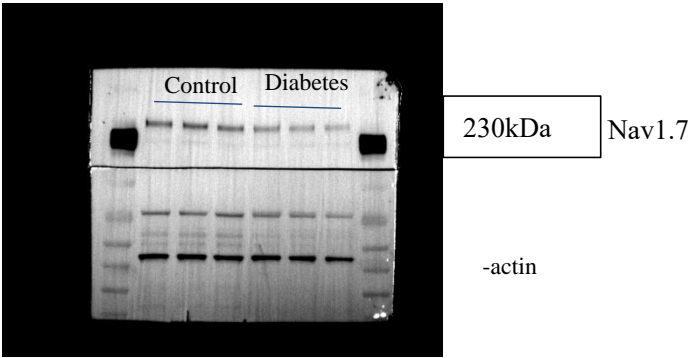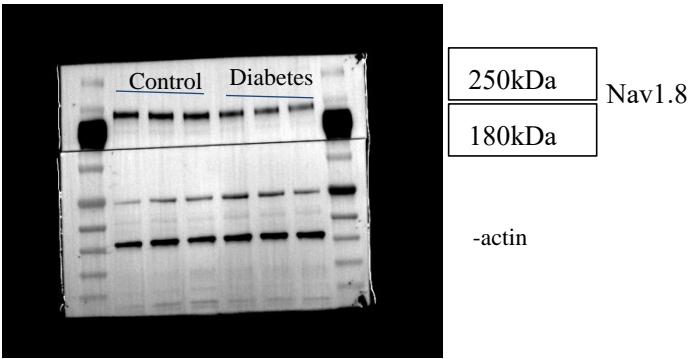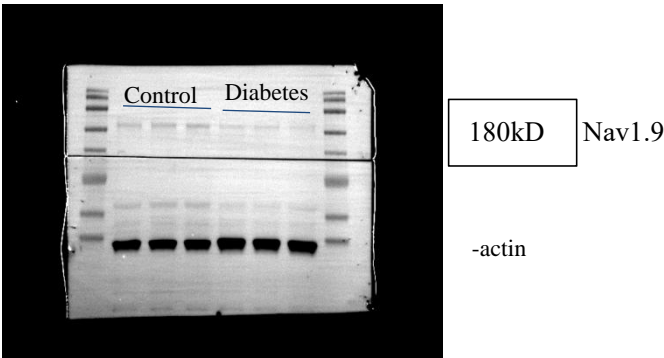

Figure 4L

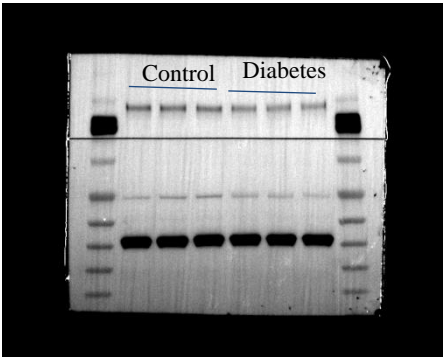

230kDa Nav1.7

-actin

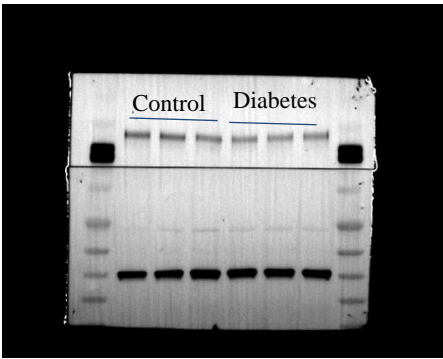

250kDa  
180kDa Nav1.8

-actin

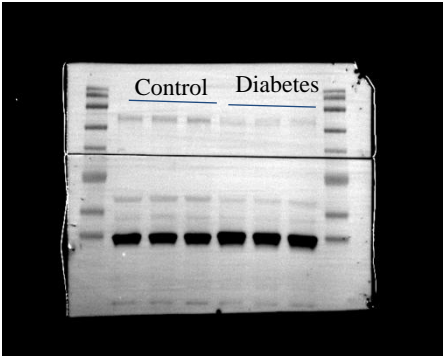

180kD Nav1.9

-actin

Figure 6H

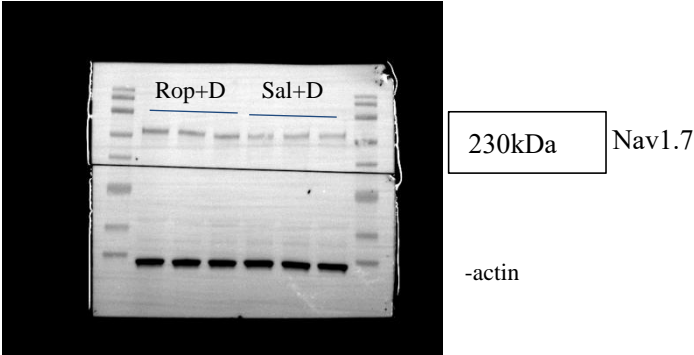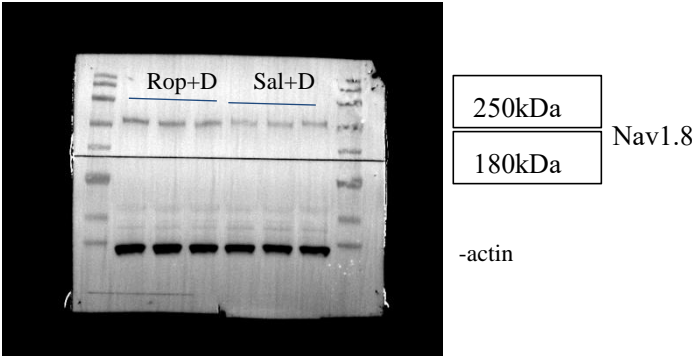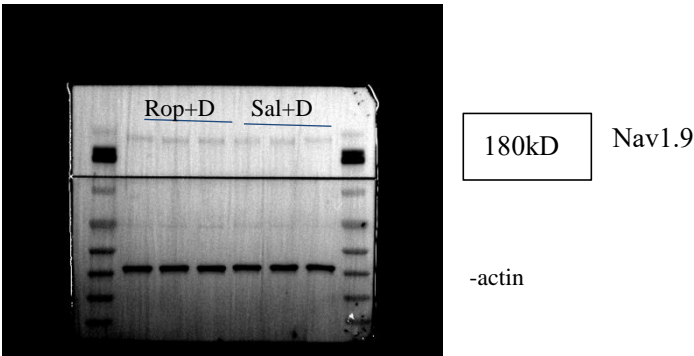

Figure 6L

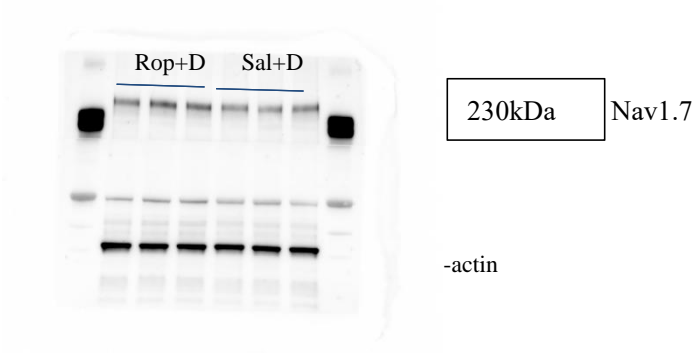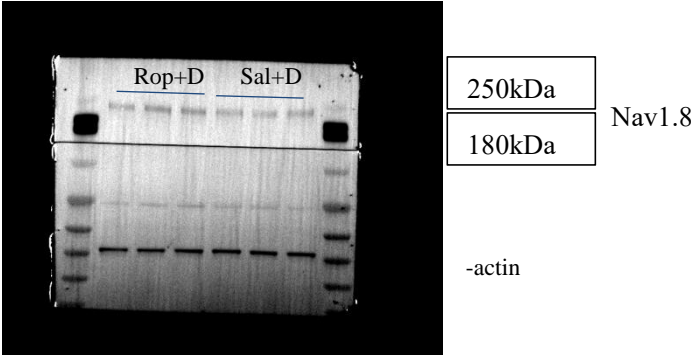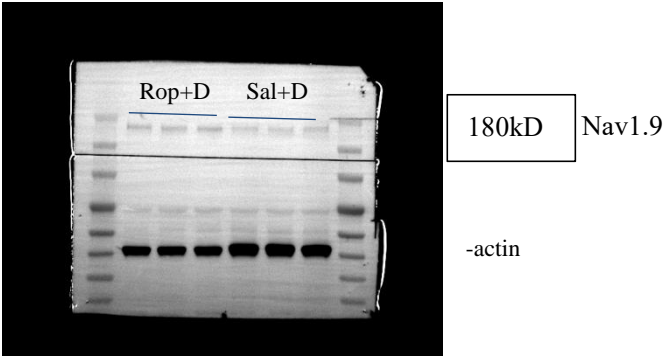

Supplement: Supplementary file 3 — Supplementary Information 3. [file 41598_2026_49845_MOESM3_ESM.pdf]
